# Restless Legs Syndrome and Its Associated Risk Factors in Parkinson's Disease

**DOI:** 10.1155/2013/535613

**Published:** 2013-12-19

**Authors:** Shahrul Azmin, Abdul Manaf Khairul Anuar, Wan Yahya Nafisah, Hui Jan Tan, Azman Ali Raymond, Othman Hanita, Shamsul Azhar Shah, Mohamed Ibrahim Norlinah

**Affiliations:** ^1^Department of Medicine, Universiti Kebangsaan Malaysia Medical Centre, Jalan Yaacob Latif, Cheras, 56000 Kuala Lumpur, Malaysia; ^2^Department of Pathology, Universiti Kebangsaan Malaysia Medical Centre, Jalan Yaacob Latif, Cheras, 56000 Kuala Lumpur, Malaysia; ^3^Department of Public Health, Universiti Kebangsaan Malaysia Medical Centre, Jalan Yaacob Latif, Cheras, 56000 Kuala Lumpur, Malaysia

## Abstract

*Introduction*. Restless legs syndrome has been shown to negatively impact the quality of life of patients. Studies have shown an association between restless legs syndrome and Parkinson's disease. We attempted to investigate the prevalence of restless legs syndrome in Parkinson's disease patients and to identify associated risk factors. *Method*. This was a cross-sectional study among patients with idiopathic Parkinson's disease. Exclusion criterion was a Mini Mental State Examination score of less than 21/30. The International Restless Legs Syndrome Study Group criterion was used to identify patients with restless legs syndrome. *Results*. A total of 113 patients were recruited. The prevalence rate of restless legs syndrome in our cohort was 9.7% and was significantly associated with a younger onset of Parkinson's disease (*P* = 0.023), male gender (*P* = 0.045), higher Mini Mental State Examination score (*P* = 0.004), and less advanced Hoehn & Yahr stage (*P* = 0.014). *Conclusion*. The prevalence rate of restless legs syndrome in our Parkinson's disease population is in keeping with other studies published worldwide. The significance of the association between a younger onset of Parkinson's disease and restless legs syndrome needs to be further investigated.

## 1. Introduction

Restless legs syndrome (RLS) is one of the most common movement disorders in the general population [1–4]. The condition has traditionally been classified as primary or secondary to other medical conditions such as low iron stores, renal failure, pregnancy, and rheumatological conditions [5–8]. Recent studies have revealed a significant genetic interplay in the development of RLS [9]. It has been shown to be one of the causes of intrinsic insomnia [10]. Left undiagnosed and untreated, RLS negatively impacts the quality of life of sufferers [11, 12].

There have been studies showing an association between RLS and Parkinson's disease (PD) [13–15]. Both of these conditions have been shown to respond to dopaminergic treatment [16]. There have been suggestions that the prevalence rate of RLS in PD patients between the Caucasian and Asian population is different, attributable to cultural and genetic differences [17].

We aim to study the prevalence of RLS in PD patients in Malaysia. We also attempted to investigate any possible risk factors that could be associated with RLS in PD patients.

## 2. Methodology

This was a cross-sectional observational study conducted at Universiti Kebangsaan Malaysia Medical Centre from December 2009 till August 2010. Consecutive PD patients aged 18 and older, attending the neurology clinic, were invited to participate in the study. The diagnosis of PD was made by a neurologist with competence in movement disorders according to the UK PD Brain Bank Criteria. Patients were excluded if they had a MMSE score of less than 21/30. The study complies with the Declaration of Helsinki and was approved by the Institution's Ethics committee.

Patients who agreed to participate and gave informed consent were interviewed and examined clinically. A semistructured interview was used to obtain information on disease history and other sociodemographic information of patients such as gender, age, disease onset, Hoehn & Yahr stage, and disease duration. The diagnosis of RLS was made using the 4 major symptoms of RLS developed by the International Restless Leg Syndrome Study Group (IRLSSG): (1) an urge to move the legs, usually accompanied or caused by uncomfortable sensation in the legs, (2) the beginning or worsening of symptoms during period of rest or inactivity, (3) the partial or total relief of symptoms by movement, and (4) the symptoms being worse in the evening or night than during the day or only occurring in the evening or night. A positive diagnosis of RLS was made when a patient had all of the four symptoms described above [18]. In addition, the severity of RLS was evaluated using the IRLSSG rating scale [19]. Either the English or the Malay version of each of the questionnaires was administered according to the patients' language preference by a single interviewer.

After the completion of the above questionnaire, 4 mLs of venous blood was drawn from the patients and analysed for haemoglobin levels, serum iron levels, serum ferritin, transferrin saturation, and total iron binding capacity (TIBC).

For analytic purposes, the total daily levodopa equivalent unit (LEU, mg/day) was calculated based on previously established methods, where 100 mg of levodopa = 130 mg of levodopa in controlled released form, 70 mg of levodopa if also using entacapone, 1 mg of pramipexole, 5 mg of ropinirole, 5 mg of rotigotine, and 100 mg of piribedil [20, 21].

All data were analysed using SPSS 18.0 statistical software package. Qualitative and quantitative demographic characteristics were tabulated and tested for normality using Shapiro-Wilk test. Results were expressed by mean ± standard deviation (SD) or median with interquartile range (IQR). To compare means of two normally distributed data, Student *t*-test was used. For nonnormally distributed data, Mann-Whitney *U* test was used to compare between two groups. For comparison of proportions between two groups, Chi-square and Fisher's exact test were used. Correlations between these variables were also assessed by Spearman's Rho test.

A *P* value of <0.05 was deemed as statistically significant.

## 3. Results 

A total of 113 patients who fulfilled the inclusion and exclusion criteria were enrolled into the study (Table 1).

The overall mean age of this study population was 64.8 ± 9.0 years. The mean age of onset of PD was 59.0 ± 9.9 years and the median duration of illness was 5.0 (2.0–8.0) years.

Seventy-one (62.8%) patients were Chinese, 32 (28.3%) were Malay, 8 (7.1%) were Indian, and 2 (1.8%) were from other ethnicities as listed previously. There were 60 (53.1%) males and 53 (46.9%) females in the study cohort. The median Hoehn & Yahr stage was 2.0 (2.0-3.0).

Eleven (9.7%) out of the 113 patients recruited in this study fulfilled the IRLSSG criteria for RLS. The median age of these patients was 59.0 years (50.0–66.0) and only 2 (18.2%) were females.

Of the patients who fulfilled the criteria for RLS, 7 (63.6%) were of Chinese ethnicity, 3 (27.3%) were Malay, and the remainder 1 (9.1%) patient was Indian.

Patients with RLS had significantly lower age of onset of PD (*P* = 0.023) and there were more males with RLS compared to females (*P* = 0.045). PD patients with RLS were at a less advanced disease stage according to the Hoehn & Yahr stage as compared to the patient without RLS (*P* = 0.014). Finally, PD patients with RLS had a significantly better MMSE score as compared to the PD patients without RLS (*P* = 0.04) (Table 2).

There was no statistically significant difference between ethnicity and duration of illness between the group of patients with RLS and without RLS manifestation.

We compared the dopaminergic medication dose between the groups with RLS and without RLS. There was no statistically significant difference in the levodopa dosage, dopamine agonist dosage, and the combined dosage of levodopa with dopamine agonist between the patients with RLS and without RLS (*P* = 0.988, *P* = 0.098, and *P* = 0.666) (Table 2).

In our study, serum ferritin concentration was lower and the transferrin saturation was higher in patients with RLS compared to those without RLS, but these findings were not statistically significant (*P* = 0.616, and *P* = 0.483) (Table 2).

## 4. Discussion

In our study, we found that the prevalence of RLS in PD patients was 9.7%. Krishnan et al., investigating RLS in PD patients in the Indian subcontinent, found a prevalence rate of 7.9% [22]. In the Far East, Nomura et al. and Lee et al., investigating RLS in PD patients in the Japanese and Korean populations, quoted a prevalence rate of 12% and 16.3%, respectively [23, 24]. Our finding on the prevalence rate of RLS in PD patients appears to be consistent with findings of previous studies.

Our study showed that patients with RLS had a younger age of onset of PD. Our findings are echoed by Peralta et al. and Nomura et al., which demonstrated the same observation [23, 25]. In contrast, Krishnan et al. and Ondo et al. found the opposite; in their cohort, patients with RLS tended to have an older age of onset of PD [13, 22]. Why this is so remains unclear. A genetic explanation, considering the different ethnic backgrounds in each study, has been proposed. Unfortunately, to date, common genetic risk variants associated with RLS have so far not been detected in PD patients [26, 27].

It has been shown that early onset Parkinson's disease is associated with certain genetic mutations such as *Parkin, LRRK2,* and *PINK1* [28, 29]. Furthermore, there have been several case reports describing RLS in patients with *Parkin, LRRK2*, and *PINK1* mutations [30–32]. It may be worth exploring whether there is any association between monogenic Parkinson's disease and RLS, as this may explain the association between younger age of onset of Parkinson's disease and RLS that we observed in our study.

We also found that, in our cohort, PD patients with RLS had a better MMSE score and were at a less advanced stage of disease as compared to PD patients without RLS. We hesitate to put too much weight into these findings as we feel this is related perhaps to the fact that, in our cohort, the PD patients with RLS tended to be younger than the PD patients without RLS, although this was not significant (59 years versus 66.5 years; *P* = 0.066).

Dopaminergic medications have been shown to have an augmentation effect on the symptoms of RLS [33, 34]. Although there was a trend towards a higher dopamine agonist dose in patients with RLS as compared to patients without RLS (*P* = 0.098), our study did not show any statistically significant difference in the dose of the dopaminergic medications between the 2 groups of patients. We wonder whether our limited sample size had any impact on the statistical analysis and whether a further study with a bigger sample size would reveal some statistically significant results. This is important as it may explain why, comparatively, our patients with RLS had a lower Hoehn & Yahr score; the higher dopaminergic dose in the patients with RLS may, directly or indirectly, reduce the severity of the patients' Parkinson's disease. Further studies with a bigger sample size are required to put this question to rest.

Iron plays a role in the biosynthesis and transmission of dopamine and dysfunctional iron storage forms the link between the pathogenesis of RLS and PD [35]. We did not find any statistically significant difference in the serum ferritin levels and transferrin saturation levels between the patients with and without RLS. Ondo et al. showed in their cohort that their PD patients with RLS had significantly lower serum ferritin levels than those without RLS [13].

Overall, current data on RLS in PD are conflicting and have only shown a chance association between RLS and PD. This is not surprising considering the fact that both disorders are common in the elderly. Furthermore, there have been no prospective studies that have assessed the risk of developing PD among identified RLS cohorts. A number of confounding factors complicate the findings of prevalence studies of RLS in PD, including dopaminergic treatment, which is known to either mask or augment any possible coexisting RLS symptoms [36]. Low ferritin levels (<50 *μ*g) were also more common in PD patients with RLS than in those without, suggesting that iron deficiency might play a predisposing role for RLS in PD [14].

Our study has strengths. Firstly, the questionnaire was administered by a single-trained personnel to address the issue of interviewer bias. Secondly, we conducted our study in a population comprised of 3 different ethnicities. Previous studies on RLS were generally performed in a more homogenous population. There has been speculation that there are differences in the prevalence of RLS in PD patients of different ethnicities [37]. We did not find any statistically significant difference along ethnic lines and our study lends itself to the increasing body of knowledge of RLS in PD patients, especially relating to ethnic differences.

Several limitations of current study have been identified. As the questionnaire involved events in the past, some elements of recall bias may have had an impact on the results of our study. The recruited patients were mainly in the early stage of Parkinson's disease and may not give a true representation of the PD population. We made every effort to be as stringently as possible in the diagnosis of RLS by strictly adhering to the IRLSSG criteria. However, this diagnostic criterion has not been validated in a PD population and the possibility of inadvertently including RLS mimics such as akathisia and other Parkinson's disease-related sensory symptoms might have occurred.

## 5. Conclusion

We found that the prevalence rate of RLS in PD patients was 9.7%, in keeping with other published studies worldwide. In our study, PD patients with RLS tended to have younger onset of PD. The significance of this is unclear and further studies are required to elucidate this question.

## Figures and Tables

**Table 1:** Baseline characteristics of study population.

	*n* = 113
Age, years, mean (SD)	64.8 (9.0)
Sex, *n* (%)	
Male	60 (53.1)
Female	53 (46.9)
Ethnicity, *n* (%)	
Malay	32 (28.3)
Chinese	71 (62.8)
Indian	8 (7.1)
Others	2 (1.8)
Duration of illness of PD, years	5 (2.0–8.0)
Levodopa dose, mg	300 (450)
LEU of dopamine agonist, mg	0 (75)
Total LEU, mg	350 (480)
Hoehn and Yahr stage	2 (2.0-3.0)
Stage 1, *n* (%)	26 (23)
Stage 2, *n* (%)	54 (48.7)
Stage 3, *n* (%)	30 (26.5)
Stage 4, *n* (%)	2 (1.8)

Data is expressed as median (IQR) unless otherwise stated.

LEU: levodopa equivalence unit; total LEU: levodopa plus LEU of dopamine agonist.

**Table 2:** Comparison of PD patients with and without RLS.

	RLS, *n* = 11	No RLS, *n* = 102	*P* value
Age, years	59 (50–66)	66.5 (59–72)	0.066
Age of PD onset, years	52 (47–60)	61 (54–66.3)	0.023
Gender, *n* (%)			
Male	9 (15)	51 (85)	0.045
Female	2 (3.8)	51 (96.2)	
Ethnicity, *n* (%)			
Malay	3 (9.7)	28 (90.3)	0.991
Chinese	7 (9.7)	65 (90.3)	
Indian	1 (9.7)	7 (87.5)	
Others	0 (0)	2 (100)	
Duration of illness of PD, years	4 (1–9)	5 (2–8)	0.876
Hoehn and Yahr stage	2 (1-2)	2 (2-3)	0.014
MMSE	30 (29-30)	28 (24–30)	0.004
Levodopa, mg	390 (750)	300 (442.5)	0.988
Dopamine agonist (LEU), mg	100 (150)	0 (50)	0.098
Total LEU, mg	390 (700)	350 (457)	0.666
Serum ferritin, ng/mL	108.3 (55.7–193.4)	131.5 (72.3–206.6)	0.616
Transferrin saturation, %	34.2 (20.2–42.1)	26.3 (21.6–32.2)	0.483
Haemoglobin, g/dL	13.4 (12.6–15.3)	13.3 (12.4–14.2)	0.399
Serum iron, ug/dL	15.4 (11.5–23.6)	13.0 (11.1–15.5)	0.132
TIBC, %	56 (47–57)	48.5 (44–54)	0.108
RLS severity, *n* (%)			
None	102 (90.3)		
Mild	4 (3.5)		
Moderate	4 (3.5)		
Severe	3 (2.7)		
Very severe	0 (0)		

Data is expressed as median (IQR) unless stated otherwise.

MMSE: mini mental state examination (range of score 0–30); PD: Parkinson's disease; RLS: restless leg syndrome; TIBC: total iron binding capacity; LEU: levodopa equivalence unit; total LEU: levodopa plus LEU of dopamine agonist.
